# Gender specific patterns of age-related decline in aortic stiffness: a cardiovascular magnetic resonance study including normal ranges

**DOI:** 10.1186/s12968-015-0126-0

**Published:** 2015-02-19

**Authors:** Richard M Nethononda, Adam J Lewandowski, Ross Stewart, Ilias Kylinterias, Polly Whitworth, Jane Francis, Paul Leeson, Hugh Watkins, Stefan Neubauer, Oliver J Rider

**Affiliations:** University Oxford Centre for Clinical Magnetic Resonance Research, Radcliffe Division of Medicine, Department of cardiovascular Medicine, Oxford, UK; Chris Hani Baragwanath Hospital, Soweto & University of Witwatersrand, Johannesburg, RSA; Oxford Cardiovascular Clinical Research Facility, University of Oxford, Oxford, UK; University of Exeter and Plymouth, Exeter, UK

**Keywords:** Aorta, Cardiovascular magnetic resonance, Age

## Abstract

**Background:**

Young females exhibit lower cardiovascular event rates that young men, a pattern which is lost, or even reversed with advancing age. As aortic stiffness is a powerful risk factor for cardiovascular events, a gender difference with advancing age could provide a plausible explanation for this pattern.

**Methods:**

777 subjects (♀n = 408, ♂n = 369) across a wide range of age (21–85 years) underwent cardiovascular magnetic resonance to assess aortic pulse wave velocity (PWV) and, in addition, aortic distensibility at three levels; 1) ascending aorta (Ao) and 2) proximal descending aorta (PDA) at the level of the pulmonary artery and 3) the abdominal aorta (DDA).

**Results:**

There was a strong negative correlation between increasing age and regional aortic distensibility (Ao♀R-0.84, ♂R-0.80, PDA♀R-0.82, ♂R-0.77, DDA♀R-0.80, ♂R-0.71 all p < 0.001) and a strong positive correlation with PWV, (♀R0.53, ♂R 0.63 both p < 0.001). Even after adjustment for mean arterial pressure, body mass index, heart rate, smoking and diabetes, females exhibited a steeper decrease in all distensibility measures in response to increasing age (Ao♀-1.3 vs ♂-1.1 mmHg-1, PDA ♀-1.2 vs ♂-1.0 mmHg, DDA ♀-1.8 vs ♂-1.4 mmHg-1 per 10 years increase in age all p < 0.001). No gender difference in PWV increase with age was observed (p = 0.11).

**Conclusion:**

Although advancing age is accompanied by increased aortic stiffness in both males and females, a significant sex difference in the rate of change exists, with females showing a steeper decline in aortic elasticity. As aortic stiffness is strongly related to cardiovascular events our observations may explain the increase in cardiovascular event rates that accompanies the menopausal age in women.

## Background

Cardiovascular disease develops between 7 to 10 years later in women than in men and is the leading cause of death and morbidity among women [1]. Although premenopausal women appear to be protected against cardiovascular disease [2], following the menopause its prevalence increases and can even exceed that observed in men [3,4]. Gender differences have traditionally been attributed to hormonal differences, particularly high oestrogen levels, which were initially speculated to confer protection to premenopausal females [4,5].

This hormonal hypothesis then lead to idea that hormonal replacement therapy (HRT) could confer continued protection against cardiovascular disease. Despite the fact that early case-controlled and observational studies suggested a potential protective role of HRT against cardiovascular disease in post-menopausal women [6-8], randomized controlled trials failed to show benefit and, even suggested a potential for harm [9-11]. This then raises the question as to what other factors, either as a result of, or in response to these hormonal changes, are responsible for the observed rapid increase in cardiovascular risk, which accompanies the menopause.

One candidate mechanism is aortic stiffening, which is not only a powerful, independent predictor of negative cardiovascular events [12-15] but also accompanies advancing age [16]. On this basis, females would be at higher cardiovascular risk than males if they showed an increased rate of stiffness with advancing age. We therefore hypothesised that age-related aortic stiffening may be different between genders and could provide a possible explanation for the increase in cardiovascular events that accompany menopause.

Cardiovascular magnetic resonance (CMR) allows direct non-invasive high resolution imaging perpendicular to the aorta, enabling measurement of size, distensibility [17] and aortic blood flow at any level [18]. The majority of PWV measures have used pressure wave analysis which has several advantages including speed, simplicity and proven clinical relevance [19]. Despite this, applanation tonometry has several limitations, including; difficulty of acquisition in obese subjects, the intimate nature of femoral pulse examination, estimation of the aortic length using surface markers and the assumption of homogeneous aortic mechanical properties with no regional differences. All of these limitations are overcome with vascular CMR assessment for both PWV and regional distensibility.

As a result, we sought to investigate 1) gender differences in age-related decline in aortic distensibility, and aortic PWV as assessed by CMR and 2) to investigate whether regional variations in age-related changes in aortic distensibility exist.

## Methods

### Study participants

The study was approved by the local research ethics committee, and informed written consent was obtained from each patient. 777 subjects (♀n = 408, ♂ n = 369) were recruited to the Oxford Families Blood Pressure Cohort [20] and to other studies within the Oxford Centre for Clinical Magnetic Resonance Research (OCMR [21,22]). All subjects underwent CMR at 1.5 T for the assessment of regional aortic distensibility and aortic PWV. History, anthropometric data, and an average of 3 clinic brachial artery blood pressure measurements were recorded.

### Inclusion criteria

All subjects were screened for the presence of identifiable cardiac risk factors and the following were documented, hypertension (defined as currently taking antihypertensive medication, or taken as an average of three supine measures over ten minutes >140/90 mmHg taken by brachial blood pressure measurement, Model, DINAMAP 1846-SX, Critikon Corp), diabetes (defined as a history of diabetes on medication, or a fasting glucose >6.5 mmol/l), smoking (current status). Subjects were excluded if they were pregnant or had given birth within 6 months, or had any contraindications to CMR. Subjects were also excluded if they had a history of valvular heart disease, were under 20 years of age (age range 20 – 80 yrs).

#### Anthropometric data

For the calculation of the waist: hip ratio, the average of three waist measurements was recorded at a) the level of the umbilicus, and b) the level of the greater trochanter of the femur as previously described [23].

#### Aortic imaging

All MR scans for the assessment of PWV and aortic distensibility were performed on a 1.5 Tesla MR system (Siemens Healthcare, Erlangen, Germany).

##### Aortic distensibility

Aortic distensibility was assessed using an SSFP cine sequence with the following parameters: TR 42 ms, TE 1.4 ms, FOV read 380 mm, in plane resolution 1.97 mm, slice thickness 7 mm. Based on sagittal-oblique pilot images aligned with the aortic arch, aortic cine images were acquired in transverse planes at 3 levels: the crossing of the pulmonary arch through 1) the ascending thoracic aorta (Ao), 2) descending thoracic aorta (PDA) and 3) 12 cm below this slice in the abdomen (DDA) as previously described [24]. The abdominal cine images were piloted perpendicular to the orientation of the abdominal aorta. During the acquisition of the images, a brachial blood pressure was recorded (DINAMAP 1 846-SX, Critikon Corp) to provide the systolic (Ps) and diastolic (Pd) aortic pressures at the same time as the aortic cross sectional area images were being acquired.

#### CMR protocol for aortic pulse wave velocity

To measure PWV, a free breathing, retrospectively ECG-gated, spoiled gradient echo sequence with velocity-encoding gradient for phase contrast was used to measure through-plane flow. Based on sagittal-oblique pilot images aligned with the aortic arch, aortic flow was acquired in same two transverse planes as the Ao and DDA distensibility measurements described above. The abdominal flow images were piloted perpendicular to the orientation of the abdominal aorta. Scan parameters were: effective TR 1 RR-interval, TE 2.8 ms, in-plane resolution 1.3 mm, slice thickness 5 mm, temporal resolution 10 ms as previously described [25].

#### Image analysis

##### Aortic distensibility

Aortic cross-sectional areas for aortic segments (Ao, PDA and DDA) in systole and diastole were calculated using semi-automated in-house software within Matlab version R2012a (Mathworks, Natick, Massachusetts) [26]. Aortic distensibility (AD) was calculated according to the formula: AD = [(Amax – Amin)/Amin)/pulse pressure], where Amax and Amin are aortic maximal systolic and minimal diastolic areas (mm^2^), respectively. All images were analysed blinded to gender and clinical details.

##### Aortic pulse wave velocity

**Figure 1:**
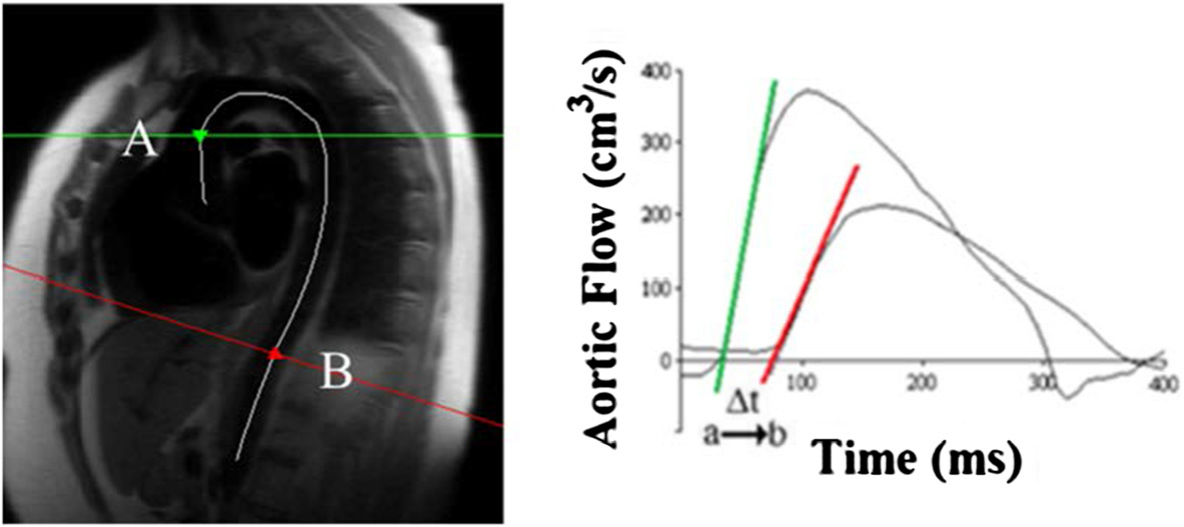
**Calculation of Pulse Wave Velocity. Distance (white line, left panel, Δx) was calculated between A (green line, ascending aorta) and B (red line, abdominal aorta).** Corresponding Aortic flow/time curves used to calculate the arrival times of aortic pulse waveform are shown in the right panel. Δt represents the time (ms) between the intercepts of the tangents to the flow in the ascending aorta (**a**, green line) and the abdominal aorta (**b**, red line). Pulse wave velocity is then calculated as Δx/Δt.

To analyse aortic PWV, aortic flow images for aortic segments (Ao, PDA and DDA) were calculated using semi-automated in-house software within Matlab version R2012a (Mathworks, Natick, Massachusetts). Aortic PWV was determined as Δx/Δt (m/s), where Δx is the aortic distance between two imaging levels and Δt is time delay between the arrival of the foot of the pulse wave between these imaging levels (Figure 1) as previously described [25].

#### Statistical analysis

All statistics were analysed using commercial software packages (SPSS 20; SPSS, Chicago, Ill, STATA, StataCorp, Texas). All data were subjected to Kolmogorov–Smirnov tests to establish normal distribution of the data. All normally distributed results are presented as the mean ± standard deviation unless otherwise stated. Age group separated, normally distributed data, were analysed using ANOVA analysis with Bonferroni correction. Linear regression analysis was also used to assess the effect of age on aortic distensibility and PWV measures within the dataset. We assessed sex differences in the effects of age and other predictors using interaction terms in the linear regression models for each outcome. Additional adjusted regression models accounting for the effects of, mean arterial pressure, heart rate and body mass index was also performed. Linearity was assessed by the addition of a quadratic term to the regression analyses. No violations of linearity were observed. Normality of distribution of the standardised errors was assessed, all standardised errors were normally distributed for the primary associations of interest. Values of p < 0.05 were considered as statistically significant.

## Results

### Baseline characteristics

Subjects were separated into groups according to gender and age (20-29 yrs ♂n = 92, ♀n = 117, 30-39 yrs, ♂n = 54, ♀n = 44, 40-49 yrs ♂n = 80, ♀n = 66, 50-59 yrs ♂n = 57, ♀n = 39, 60-69 yrs ♂n = 56, ♀n = 61 and >70 yrs ♂n = 57, ♀n = 51). As expected, increasing age was associated with an increase in systolic blood pressure (R 0.45, p < 0.001), BMI (R 0.12, p < 0.001), diabetes (R 0.12, p < 0.02) and hypercholesterolaemia (R 0.29, p < 0.001, Table 1). Importantly, all male and female age groups were well matched for body mass index, systolic blood pressure, diastolic blood pressure, smoking status, hypercholesterolaemia, presence of diabetes (all p > 0.05, Table 1) and medications (Table 2).

**Table 1:** **Baseline characteristics of the study group separated in age groups**

	**Age groups**											
	**20-29**		**30-39**		**40-49**		**50-59**		**60-69**		**>70**	
	**Male**	**Female**	**Male**	**Female**	**Male**	**Female**	**Male**	**Female**	**Male**	**Female**	**Male**	**Female**
	**(n = 92)**	**(n = 117)**	**(n = 54)**	**(n = 44)**	**(n = 66)**	**(n = 80)**	**(n = 39)**	**(n = 57)**	**(n = 61)**	**(n = 56)**	**(n = 57)**	**(n = 54)**
**Age (years)**	25 ± 2	26 ± 2	35 ± 3	35 ± 3	45 ± 3	45 ± 3	54 ± 3	54 ± 3	65 ± 3	65 ± 3	76 ± 4	75 ± 4
**BMI (kg/m** ^**2**^ **)**	22 ± 7	23 ± 7	28 ± 7	26 ± 7	29 ± 5	28 ± 7	29 ± 7	29 ± 6	28 ± 4	28 ± 4	28 ± 3	30 ± 10
**SBP (mmHg)**	111 ± 30	110 ± 26	107 ± 40	112 ± 23	132 ± 16	124 ± 15	134 ± 15	133 ± 19	142 ± 16	143 ± 15	149 ± 20	150 ± 15
**DBP (mmHg)**	65 ± 18	69 ± 16	65 ± 25	76 ± 14	85 ± 9	76 ± 9	85 ± 10	80 ± 10	82 ± 10	81 ± 9	79 ± 11	79 ± 8
**Waist: Hip ratio**	0.85 ± 0.05	0.78 ± 0.07	0.9 ± 0.07	0.81 ± 0.07	0.99 ± 0.06	0.86 ± 0.09	0.98 ± 0.10	0.87 ± 0.71	0.97 ± 0.06	0.85 ± 0.06	0.99 ± 0.06	0.87 ± 0.07
**% Smoking**	3	4	7	4	20	15	35	30	38	34	42	37
**% High Cholesterol**	0	0	2	0	3	0	5	2	10	5	23	25
**% Diabetic**	0	0	0	0	2	0	5	2	2	0	5	11

**Table 2:** **Medications for the study group**

	**Age Groups**											
	**20-29**		**30-39**		**40-49**		**50-59**		**60-69**		**>70**	
	**Male**	**Female**	**Male**	**Female**	**Male**	**Female**	**Male**	**Female**	**Male**	**Female**	**Male**	**Female**
	**(n = 92)**	**(n = 117)**	**(n = 54)**	**(n = 44)**	**(n = 66)**	**(n = 80)**	**(n = 39)**	**(n = 57)**	**(n = 61)**	**(n = 56)**	**(n = 57)**	**(n = 54)**
**Aspirin (%)**	0.0	0.0	0.0	0.0	3.0	1.3	7.7	7.0	24.6	10.7	28.1	31.5
**Beta Blocker (%)**	0.0	0.0	1.9	2.3	3.0	1.3	7.7	7.0	11.5	14.3	24.6	20.4
**Calcium Channel Blocker (%)**	0.0	0.0	1.9	0.0	4.5	3.8	10.3	3.5	23.0	16.1	29.8	33.3
**ACE inhibitor/ARB (%)**	0.0	0.0	3.7	0.0	7.6	11.3	15.4	14.0	36.1	37.5	40.4	40.7
**Diuretic (%)**	0.0	0.0	0.0	2.3	3.0	2.5	2.6	10.5	13.1	16.1	22.8	27.8
**Statin (%)**	0.0	0.0	1.9	0.0	0.0	1.3	10.3	3.5	21.3	14.3	28.1	27.8
**HRT (%)**	0.0	0.0	0.0	0.0	0.0	0.0	0.0	1.8	0.0	3.6	0.0	3.7
**Taking Medication (%)**	0.0	0.0	5.6	2.3	10.6	12.5	28.2	29.8	65.6	58.9	80.7	75.9

#### The effect of aging on aortic stiffness

##### Aortic distensibility

**Figure 2:**
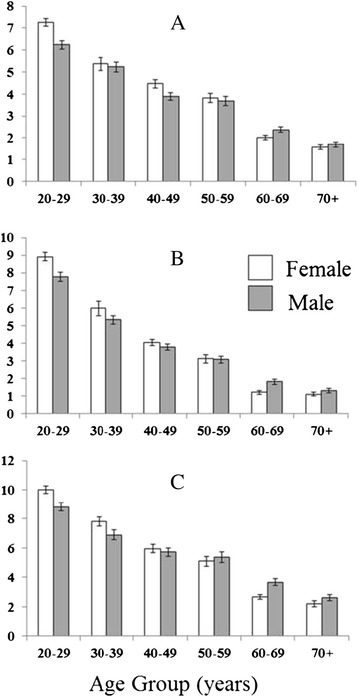
**Gender specific effects of age on regional aortic distensibility measured in the (A) ascending aorta, (B) the proximal descending aorta and (C) the abdominal aorta.**

**Figure 3:**
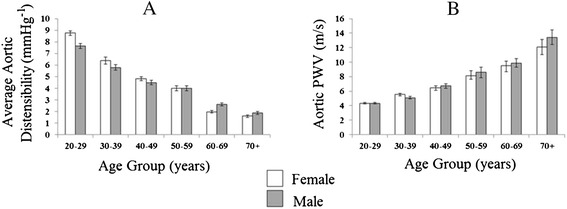
**Gender specific effects of age on (A) average aortic distensibility and (B) aortic pulse wave velocity.**

As expected, in both sexes, there was a strong negative correlation between age and all measures of regional aortic distensibility (Ao ♂R-0.80, ♀R-0.84, PDA, ♂R-0.77, ♀R-0.82, DDA ♂R −0.71, ♀R-0.80, all p < 0.001, Figure 2, Table 3). A similar pattern was seen with average aortic distensibility (♂R −0.80, ♀R-0.85, p < 0.001, Figure 3). In addition to age, all aortic distensibility measures were negatively correlated with mean arterial blood pressure (R-0.55-0.58, all p < 0.001), body mass index (R-0.33 to −0.38, all p < 0.001) heart rate (R-0.13 to −0.16, all p < 0.01), smoking status (R-0.25 to −0.30, all p < 0.001) and diabetes (R-0.09 to −0.10, all p < 0.02).

**Table 3:** **Aortic characteristics of the study group separated in age groups**

	**Age groups**											
	**20-29**		**30-39**		**40-49**		**50-59**		**60-69**		**>70**	
	**Male**	**Female**	**Male**	**Female**	**Male**	**Female**	**Male**	**Female**	**Male**	**Female**	**Male**	**Female**
	**(n = 92)**	**(n = 117)**	**(n = 54)**	**(n = 44)**	**(n = 66)**	**(n = 80)**	**(n = 39)**	**(n = 57)**	**(n = 61)**	**(n = 56)**	**(n = 57)**	**(n = 54)**
**Pulse Wave Velocity (m/s)**	4.4 ± 0.8	4.3 ± 0.7	5.1 ± 1.0	5.5 ± 1.6	6.8 ± 2.3	6.5 ± 3.1	8.6 ± 4.4	7.8 ± 2.8	9.6 ± 4.1	9.6 ± 6.4	13.0 ± 7.7	11.9 ± 7.5
**Aortic Distensibility (mmHg** ^**−1**^ **)**												
**Ascending**	7.8 ± 2.5	8.9 ± 2.5	5.3 ± 1.8	5.9 ± 2.6	3.8 ± 1.3	4.0 ± 1.6	3.3 ± 1.6	3.1 ± 1.8	1.8 ± 1.3	1.2 ± 0.8	1.3 ± 0.9	1.1 ± 0.8
**Proximal Descending**	6.2 ± 1.7	7.1 ± 1.9	5.3 ± 1.8	5.2 ± 2.0	3.9 ± 1.4	4.5 ± 1.7	3.7 ± 1.3	3.8 ± 1.5	2.4 ± 1.0	2.0 ± 0.8	1.7 ± 0.9	1.6 ± 0.7
**Abdominal**	8.8 ± 2.6	9.9 ± 2.9	6.9 ± 2.4	7.7 ± 2.1	5.7 ± 2.2	5.9 ± 2.4	5.3 ± 2.1	5.1 ± 2.4	3.7 ± 1.7	2.7 ± 1.2	2.6 ± 1.6	2.2 ± 1.4

Importantly, in females, the largest decrease in all measures of regional aortic distensibility occurred between age group 50–59 (54 ± 3 years) and age group 60–69 (65 ± 3 years). Although the groups were separated by an increase of only 11 years a 47-61% decrease in distensibility was observed (Ao by 61%, PDA by 47%, DDA by 47%, all p < 0.001, Table 3). In comparison, in males, although the largest decrease in distensibility also occurred between the age groups 50–59 (54 ± 3 years) and 60–69 (65 ± 3 years) only a 31-45% decrease in distensibility was observed (Ao by 45%, PDA by 35%, DDA by 31%, all p < 0.001, Table 3). Put together, these aortic distensibility data suggest that not only is there a general decline in aortic elastic function with increasing age, but also that, in women, the largest decrease occurs over the menopausal period.

##### Pulse wave velocity

There was a strong positive correlation between PWV and age in males (R0.63, p < 0.001, Figure 3) and females (♀R0.53, p < 0.001). PWV in the youngest male age group 20–29 was 18% lower than that recorded in the second youngest male age group 30–39 (PWV 4.3 ± 1.1 vs 5.1 ± 1.0 m/s, p < 0.001). When compared to the lowest age group, aortic PWV was increased by 54%, 98%, 128% and 209% for the next advancing age groups respectively (all p < 0.001). PWV in the youngest female age group was 29% lower than that recorded in the second youngest female age group (4.3 ± 0.9 vs 5.5 ± 1.5 m/s, p < 0.001). When compared to the lowest age group, aortic PWV was increased by 51%, 89%, 122% and 181% for the next advancing age groups respectively (all p < 0.001).

#### Gender differences in the effect of aging on aortic stiffness

##### Regional aortic distensibility

Although increasing age was associated with a decrease in aortic distensibility at all levels measured (Figures 2 and 3), the pattern of change with increasing age shows significant gender differences. Whereas female aortic distensibility measures at all levels were higher than males in the youngest age group 20-29 yrs (by 13-16%, p < 0.01, Figures 2 and 3), this pattern was reversed in the older age groups where aortic distensibility in females, at all levels was significantly lower than in males (age group 60–69, by 15- 33%, all p < 0.03). As a result, and in agreement with this, when comparing the coefficient of regression between age and distensibility in males and females, females showed a greater decrease in all distensibility measures in response to increasing age, at all levels measured (Ao♀-1.3 vs ♂-1.1 mmHg-1, PDA ♀-1.1 vs ♂-0.9 mmHg, DDA ♀-1.5 vs ♂-1.0 mmHg-1 per 10 years increase in age all p < 0.001, Figure 2).

As mean arterial pressure, body mass index, heart rate, smoking status and diabetes status all significantly correlated with regional distensibility, an adjusted model was used accounting for this was performed. This showed that the gender difference remained statistically significant (Ao♀-1.3 vs ♂-1.1 mmHg-1, PDA ♀-1.2 vs ♂-1.0 mmHg, DDA ♀-1.8 vs ♂-1.4 mmHg-1 per 10 years increase in age all p < 0.001). Put together this suggests that the decline in aortic elastic function that accompanies advancing age is greater in females than in males, and is especially marked in age groups over 60 years of age.

##### Aortic pulse wave velocity

Although increasing age was associated with increased aortic PWV across both genders (Figures 2), when comparing males and females, there was no difference in the steepness of the increase in PWV with increasing age (♀ + 1.4, ♂ + 1.7 m/s per 10 years increase in age, p = 0.11). When adjusting for the effects of mean arterial pressure, body mass index, heart rate, smoking status and diabetes status, this remained unchanged (♀ + 1.3, ♂ + 1.5 m/s per 10 years increase in age, p = 0.13).

### Measures of adiposity distribution

In order to gain an insight into the change in fat mass distribution that occurred during the menopausal period and compare this to men, BMI and waist hip ratio were compared from recordings made 13 years earlier in age groups 50-59 yrs and 60-69 yrs. Data was available for 78% of females and 79% of males. This showed that in females, an age increase of 13 years (average 47 ± 6 to 60 ± 6 yrs) was associated with a greater increase in both BMI (♀ + 1.9 vs ♂ 1.1 kg/m^2^_,_ p < 0.001) and weight (♀5.4 kg vs ♂3.6 kg, p < 0.001). Waist: hip ratio also increased in females from 0.81 ± 0.08 to 0.86 ± 0.07, p < 0.001 over this 13 year period. Overall this suggests that not only is weight gain significantly greater (by 50%) in women than men over the pre to post-menopausal period, but also that women experience an increase in waist: hip ratio. This is in keeping with both a greater weight gain and with a change to android fat deposition over the menopausal period.

## Discussion

In this study, we investigated the impact of gender on age-related decline in aortic distensibility and PWV quantified with vascular CMR. In agreement with previously published data, [27] irrespective of gender, we have shown that increasing age is associated with increasing aortic stiffness as assessed by both aortic PWV and regional aortic distensibility. Despite this overall pattern, we have shown that a significant gender specific effect of age exists, with females showing a steeper decline in all measures of aortic distensibility with advancing age. Given the link between aortic stiffness and cardiovascular events, this observation provides a plausible explanation for the loss of the protection conferred by young age in women.

### The effects of traditional risk factors in age related decline

The prevalence of cardiovascular disease can be to a large extent explained by the presence of the traditional cardiovascular risk factors including diabetes, hypertension, dyslipidaemia and smoking. As expected, the prevalence of these traditional risk factors increased with increasing age and therefore would be expected to result in increasing aortic stiffness in general with advancing age. Interestingly however, we have shown here that despite the fact that males and females were well matched for these traditional risk factors, across all age groups, a gender difference in decline in aortic elastic function remains present, even when adjusting for these risk factors. This suggests that other factors may be responsible for the observed age-related differences in distensibility between males and females. One candidate mechanism that has emerged in the literature to explain this is body weight changes that occur following the menopause which change from a gynoid pattern of fat distribution to a more android pattern. Interesting this data again shows that the menopausal period is associated with greater weight gain than the corresponding age increase in men, and also with an increased waist: hip ratio suggesting an excess deposition of abdominal fat in a more android pattern. As this age group is associated with a reversal of the pattern on aortic distensibility, with measures in females becoming lower than that in males, this may well be, at least in part responsible for the more rapid decline in aortic elastic function that seems to accompany the menopausal period.

### The effects of endogenous oestrogens on vascular function

Aging is associated with significant vascular remodeling including endothelial dysfunction, enhanced growth of intimal smooth muscle cells, and an increase in prevalence of vascular plaques [28]. In premenopausal women, it is possible that endogenous oestradiol may abrogate this adverse age-related vascular remodeling [28], by inhibiting including smooth muscle cell proliferation, endothelial dysfunction, lowering cholesterol and improving vascular tone [29]. Although it is difficult to separate vascular changes due to aging from those due to menopause, comparisons between men versus age-matched postmenopausal women and between age-matched premenopausal versus postmenopausal women suggest that endogenous estradiol delays the onset of cardiovascular events and vascular remodeling [30]. In support of this, we have shown here that, in females, not only all measures of aortic distensibility are most significantly decreased in the decade after the mean menopausal age (by up to 61%) but also that the decline at this age is greater in women than men. Put together it is likely that the removal of endogenous oestrogens from the circulation is playing a role in the rapid decline in distensibility seen here in this study in the decade after the menopause.

### Comparison with previous studies

Previous evidence for age-related differences in aortic function has been derived from ultrasound studies using Doppler blood-velocity detectors to measure aortic compliance [31]. In general, higher values of elasticity have been shown in younger females compared to age matched males [32] with a subsequent rapid decline in females between the ages of 45 and 50 years [31]. In agreement with this younger females have been shown to have higher distensibility index of the ascending aorta than males, with reversal of this pattern among the older age group [33]. Using the inherent advantage that CMR brings in allowing image acquisition in at several reproducible aortic levels, we have shown that gender differences in age-related decline in distensibility is not limited to the ascending aorta but also seen in the more distal regions of the proximal descending and abdominal aorta.

### Pulse wave velocity and age

Although this study has showed an increase in PWV with age in both men and women, no gender difference in age-related increase in aortic PWV was observed, a pattern that is reflected throughout the literature [34,35]. One previous study using phase contrast flow to investigate PWV across age found very similar result to these with a general increase in PWV with age, a pattern we have reproduced in a much larger group [35]. The reasons behind this lack of gender difference is likely to be explained by the mathematical relationship between distensibility and PWV, which is determined by the Bramwell-Hill equation; PWV = (ρx Distensibiltity)^‐ 1/2^, with ρ being blood density. As blood density increases markedly with menopause [36], it is unsurprising that the large decrease in aortic distensibility (between 47-61%) seen in this study when comparing pre-menopausal (age group 4, average age 54 yrs) to post-menopausal women (age group 5 average, age 65 yrs) is not accompanied by a similarly large increase in aortic PWV. Hence the change in rheological properties of blood accompanying the menopause may very well explain the lack of gender difference in observed aortic PWV that is evident in regional aortic distensibility.

### Limitations

This study is a post-hoc cross-sectional study and so the actual effect of aging within an individual on aortic stiffness is not possible to determine.

Whilst a gender difference is observed in this population with aortic distensibility, this data must be considered in light of a possible survivorship bias, with the imaging phenotype of the older populations of both sexes not being a true reflection of the aortic stiffness observed in those that were by definition more susceptible to cardiovascular events.

Although a history of renal dysfunction was excluded by medial history, no formal evaluation of renal function was performed in this cohort.

This study has used brachial pulse pressure assessment for determination of aortic distensibility and not central blood pressure [37]. However, as we are investigating the pattern of change with age, and not the absolute values, this would not be expected to be different using a derived central blood pressure.

This cohort was exclusively Caucasian which limits its applicability to other populations which are known to have different distensibility measurements [27]. Imaging of the ascending aortic region is subject to the effects of through plane motion as a result of ventricular contraction. This study did not account for this in the image acquisition. Menopausal status is not recorded in the study. As a result the data can only represent changes that occur in the study population over the average age of the menopausal period.

## Conclusion

Advancing age is accompanied by a stepwise increase in aortic stiffness in both males and females. When comparing men and women, there is a steeper decline in aortic stiffness in women, which is most pronounced after the age of menopause. As a result of these findings we speculate that the accelerated age-related decline of aortic elasticity in women could contribute to the relative increase in negative cardiovascular vascular events that occurs in postmenopausal women.
